# Cross-ethnic meta-analysis identifies association of the *GPX3-TNIP1* locus with amyotrophic lateral sclerosis

**DOI:** 10.1038/s41467-017-00471-1

**Published:** 2017-09-20

**Authors:** Beben Benyamin, Ji He, Qiongyi Zhao, Jacob Gratten, Fleur Garton, Paul J. Leo, Zhijun Liu, Marie Mangelsdorf, Ammar Al-Chalabi, Lisa Anderson, Timothy J. Butler, Lu Chen, Xiang-Ding Chen, Katie Cremin, Hong-Weng Deng, Matthew Devine, Janette Edson, Jennifer A. Fifita, Sarah Furlong, Ying-Ying Han, Jessica Harris, Anjali K. Henders, Rosalind L. Jeffree, Zi-Bing Jin, Zhongshan Li, Ting Li, Mengmeng Li, Yong Lin, Xiaolu Liu, Mhairi Marshall, Emily P. McCann, Bryan J. Mowry, Shyuan T. Ngo, Roger Pamphlett, Shu Ran, David C. Reutens, Dominic B. Rowe, Perminder Sachdev, Sonia Shah, Sharon Song, Li-Jun Tan, Lu Tang, Leonard H. van den Berg, Wouter van Rheenen, Jan H. Veldink, Robyn H. Wallace, Lawrie Wheeler, Kelly L. Williams, Jinyu Wu, Xin Wu, Jian Yang, Weihua Yue, Zong-Hong Zhang, Dai Zhang, Peter G. Noakes, Ian P. Blair, Robert D. Henderson, Pamela A. McCombe, Peter M. Visscher, Huji Xu, Perry F. Bartlett, Matthew A. Brown, Naomi R. Wray, Dongsheng Fan

**Affiliations:** 1 Queensland Brain Institute, The University of Queensland, Brisbane, Queensland 4072 Australia; 2 Institute for Molecular Bioscience, The University of Queensland, Brisbane, Queensland 4072 Australia; 3 Department of Neurology, Peking University, Third Hospital, No. 49, North Garden Road, Haidian District, 100191 Beijing, China; 4 University of Queensland Diamantina Institute, The University of Queensland, Translational Research Institute, Brisbane, Queensland 4102 Australia; 5 Institute of Health and Biomedical Innovation, Queensland University of Technology, Translational Research Institute, Brisbane, Queensland 4102 Australia; 6 Department of Basic and Clinical Neuroscience, Maurice Wohl Clinical Neuroscience Institute, King’s College London, London, WC2 R2LS UK; 7 Laboratory of Molecular and Statistical Genetics and State Key Laboratory of Developmental Biology of Freshwater Fish, College of Life Sciences, Hunan Normal University, Changsha, 410081 Hunan China; 8 Department of Global Biostatistics and Data Science, School of Public Health and Tropical Medicine, Center for Bioinformatics and Genomics, Tulane University, 1440 Canal St, Suite 2001, New Orleans, LA 70112 USA; 9 Department of Neurology, Royal Brisbane & Women’s Hospital, Brisbane, Queensland 4029 Australia; 10 Department of Biomedical Sciences, Faculty of Medicine and Health Sciences, Macquarie University, Sydney, New South Wales 2109 Australia; 11 Center of System Biomedical Sciences, University of Shanghai for Science and Technology, 334, Jungong Road, Yangpu District, 200093 Shanghai, China; 12 Kenneth G. Jamieson Department of Neurosurgery, Royal Brisbane & Women’s Hospital, Herston, Queensland 4029 Australia; 13 Division of Ophthalmic Genetics, Laboratory for Stem Cell and Retinal Regeneration, The Eye Hospital of Wenzhou Medical University, 325027 Wenzhou, China; 14 Institute of Genomic Medicine, Wenzhou Medical University, 325027 Wenzhou, China; 15 Department of Rheumatology and Immunology, Shanghai Changzheng Hospital, The Second Military Medical University, 200003 Shanghai, China; 16 School of Biomedical Sciences, The University of Queensland, Brisbane, Queensland 4072 Australia; 17 Stacey MND Laboratory, Discipline of Pathology, Brain and Mind Centre, The University of Sydney, Sydney, New South Wales 2006 Australia; 18 The Centre for Advanced Imaging, The University of Queensland, Brisbane, Queensland 4072 Australia; 19 Department of Medicine, Faculty of Medicine and Health Sciences, Multidisciplinary Motor Neurone Disease Clinic, Macquarie University, Sydney, New South Wales 2109 Australia; 20 Centre for Healthy Brain Ageing, School of Psychiatry, Faculty of Medicine, The University of New South Wales, Sydney, New South Wales 2052 Australia; 21 Neuropsychiatric Institute, Prince of Wales Hospital, Randwick, New South Wales 2031 Australia; 22 Department of Neurology, Brain Center Rudolf Magnus, University Medical Center Utrecht, 3584 CG Utrecht, The Netherlands; 23 Institute of Mental Health, The Sixth Hospital, Peking University, 100191 Beijing, China; 24 Key Laboratory of Mental Health, Ministry of Health & National Clinical Research Center for Mental Disorders, Peking University, 100191 Beijing, China; 25 UQ Centre for Clinical Research, The University of Queensland, Royal Brisbane & Women’s Hospital, Brisbane, Queensland 4029 Australia

## Abstract

Cross-ethnic genetic studies can leverage power from differences in disease epidemiology and population-specific genetic architecture. In particular, the differences in linkage disequilibrium and allele frequency patterns across ethnic groups may increase gene-mapping resolution. Here we use cross-ethnic genetic data in sporadic amyotrophic lateral sclerosis (ALS), an adult-onset, rapidly progressing neurodegenerative disease. We report analyses of novel genome-wide association study data of 1,234 ALS cases and 2,850 controls. We find a significant association of rs10463311 spanning *GPX3-TNIP1* with ALS (*p* = 1.3 × 10^−8^), with replication support from two independent Australian samples (combined 576 cases and 683 controls, *p* = 1.7 × 10^−3^). Both *GPX3* and *TNIP1* interact with other known ALS genes (*SOD1* and *OPTN*, respectively). In addition, *GGNBP2* was identified using gene-based analysis and summary statistics-based Mendelian randomization analysis, although further replication is needed to confirm this result. Our results increase our understanding of genetic aetiology of ALS.

## Abstract (web summary)

Amyotrophic lateral sclerosis (ALS) is a rapidly progressing neurodegenerative disease. Here, Wray and colleagues identify association of the *GPX3-TNIP1* locus with ALS using cross-ethnic meta-analyses.

## Introduction

For people of European ancestry, the lifetime risk of amyotrophic lateral sclerosis (ALS) is 0.3–0.5%^1, 2^, with peak age of onset of 58–63 years^3^, and median survival of 2–4 years^4^. Investigations of families with multiple affected individuals have led to the identification of mutations that segregate with disease in a number of genes, including *SOD1*, *C9orf72*, *TARDBP*, *FUS* and *TBK1* ^5, 6^. However, about 90% of cases^5^ (‘sporadic ALS’ (sALS)) present with sparse or no family history. Nonetheless, genome-wide association studies (GWAS) have provided direct evidence of a genetic contribution to sALS, with estimates that ~8.5%^7^ of variance in liability is tagged by common single-nucleotide polymorphisms (SNPs). Currently, only a small proportion of this variation (0.2% of variance in liability)^7^ is accounted for by the six common loci (*C9orf72*, *UNC13A*, *SARM1*, *MOBP*, *SCFD1*, *C21orf2*) identified as significant based on association analysis of 12,577 cases and 23,475 controls^7^. The SNP-heritability estimate implies that more risk loci will be detected with increasing sample size, as found for other complex genetic diseases^8^. Whole-exome sequencing (WES) studies, designed to identify genes enriched for rare variants, have also been conducted for sALS. The largest study, comprising 2,874 cases and 6,405 controls, identified *TBK1* as a novel ALS risk gene^6^, with GWAS support for association of common loci (*p* = 6.6 × 10^−8^)^7^. Rare variant burden analysis in a WES of 1,022 index familial cases identified p.Arg261His in *NEK1* as an ALS associated variant, and follow-up in large samples suggest that this variant together with *NEK1* loss of function mutations account for ~3% of ALS cases^9^.

To date, the largest genetic studies for ALS are in the subjects of European ancestry, but common variants associated with disease are likely to be ancient and shared across ethnicities. Given sufficient power, cross-ethnic genetic studies can aid fine mapping of disease loci, exploiting differences in allele frequency and linkage disequilibrium (LD). In China, the lifetime risk of ALS is estimated to be lower (0.1%)^1^ and its mean age of onset is estimated to be a few years earlier than in Europe^4, 10^. High penetrance mutations in known ALS genes identified in Europeans have been detected in Chinese cases^11^, but the frequency of the *C9orf72* expansion is much lower (0.3%)^12^ than in Europeans (frequency 7%)^5^, and it may have arisen on a different haplotype background^12^.

In a cross-ethnic meta-analysis of the largest GWAS for ALS in Europeans^7^, together with a new Chinese data set, we identify the *GPX3-TNIP1* locus to be significantly associated with ALS (*p* = 1.3 × 10^−8^). This association is replicated in two independent Australian cohorts with a combined *p*-value of 1.7 × 10^−3^. Previous studies indicate functional relevance of both *GPX3* and *TNIP1* ^13–18^. The identification of this locus contributes to a better understanding of the genetic aetiology of ALS.

## Results

### Genome-wide association analysis

We conduct a genome-wide (GW) association analysis in a Chinese sample of 1,234 sALS cases and 2,850 controls (Supplementary Table 1 and Supplementary Figs 1−3). The genomic inflation factor *λ*_GC_ of 1.02 and *λ*_1000_ of 1.01 showed no evidence for inflation in test statistics. The combined effects of all common genetic variants on ALS liability (SNP-heritability) estimated from the Chinese GWAS data is 15.1% (SE): 4%; *p* = 9.5 × 10^−5^) using GCTA-GREML^19^ and 15.0% (SE: 3.5%) using LD score regression^20^ (intercept 1.0, which also shows no evidence of population stratification). Given the SE, these estimates are not different from the estimate of 8.5% (SE 0.5%) from European data^7^. Partitioning of the SNP-heritability by chromosome showed a significant positive correlation with chromosome length (Supplementary Fig. 4a) consistent with a polygenic architecture. Based on minor allele frequency (MAF) bin, the SNP-heritability was attributed to SNPs across the MAF range, but SEs per bin were large (Supplementary Fig. 4b); similar analyses based on European data suggested that less common SNPs tagged more variation than other MAF classes^7^.

No individual SNPs passed the GW significant *p* value threshold of 5 × 10^−8^, and none of the significant SNPs reported in the European^7^ GWAS replicated in our samples (*p* > 0.05). We also checked for the associations of two GW significant SNPs in previous GWAS of Chinese cohort of ALS patients^21^. However, we could not replicate the association in that study. We note that despite evidence for population stratification, principal components derived from SNP data of the previous study were not included as covariates in their association analysis. The *p* values for rs6703183 and rs8141797 are 0.07 and 0.12 in our Chinese samples and 0.66 and 0.94 in European GWAS results, respectively. Direction of effect sign tests (Supplementary Table 2) and polygenic risk scoring analyses (Supplementary Fig. 5) provided no conclusive evidence of shared risk loci (Nagelkerke’s *R* ^2^ = 0.002; *p* = 0.01). These results are not unexpected given the size of our sample and effect sizes estimated in Europeans. The Chinese GWAS sample had 80% power to identify common genetic variants of genotype relative risk of 1.4 and 1.8 for risk allele frequency of 0.2 and 0.05, respectively, at the GW threshold of significance *p* = 5 × 10^−8^.

### Meta-analysis

**Fig. 1:**
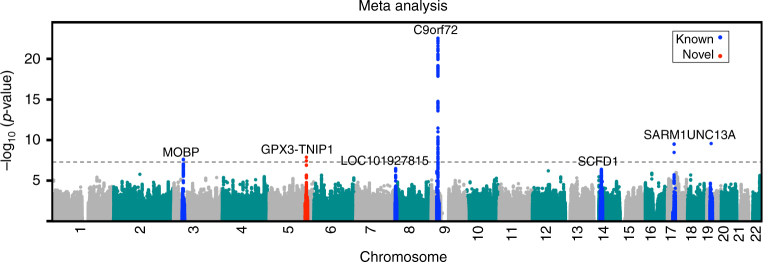
Manhattan plot of the meta-analysis between European and Chinese GWAS revealed a novel locus, *GPX3-TNIP1* (*red*). Loci previously identified in the largest European GWAS are presented in *blue*. The *p* values are from the linear mixed model

**Fig. 2:**
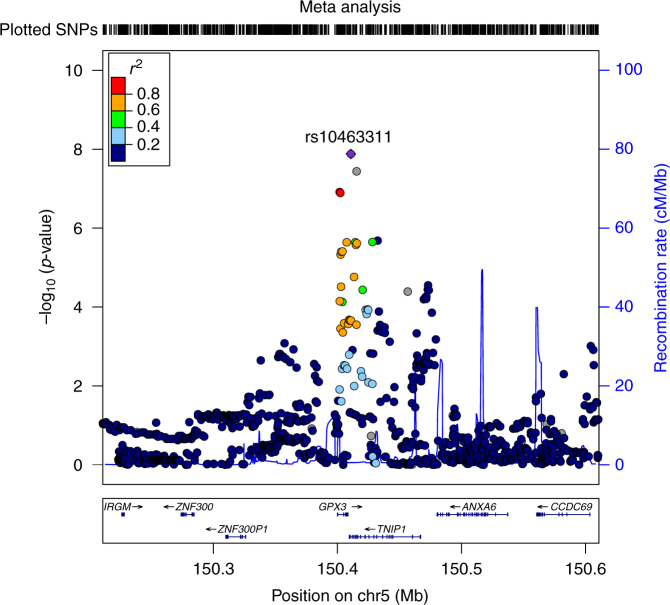
Regional ALS association plot of the *GPX3-TNIP1* locus from the meta-analysis results created using LocusZoom^44^. From the meta-analysis, rs10463311 is the SNP with the strongest association with ALS (*p* = 1.3 × 10^−8^). This SNP is replicated in two independent Australian cohorts with combined *p* = 1.7 × 10^−3^

Meta-analysis of our results with those of the European^7^ GWAS identified a new GW significant locus at chromosome 5p33.1 (rs10463311, risk allele C, odds ratio (OR) 1.11 95% confidence interval (CI): 1.06–1.14, *p*_logistic_ = 2.9 × 10^−8^; *p*_lmm_ = 1.3 × 10^−8^) spanning the genes *GPX3* and *TNIP1* (Figs. 1 and 2; Table 1; Supplementary Data 1) for which the risk allele is more common in Chinese than in Europeans (0.46 vs. 0.25). The association result was replicated in an independent Australian sample (145 cases, 116 controls, OR = 1.66; 95% CI: 1.16–2.38; *p* = 5.8 × 10^−3^) and had the same direction of effect in a second Australian sample (431 cases, 567 controls, OR = 1.22; 95% CI: 1.00–1.48; *p* = 6.2 × 10^−2^), giving a combined replication OR of 1.32 (95% CI: 1.11–1.58; *p* = 1.7 × 10^−3^) (Table 1).

**Table 1:** Association analysis results between rs10463311 spanning *GPX3-TNIP1* and ALS across cohorts

**Cohort**	***N*** **cases**	***N*** **cont**	**Freq cases**	**Freq cont**	**OR**	**95% CI**	***p***_**logistic**_
European^2^	12,577	23,475	0.27	0.24	1.11	1.07–1.15	8.5 × 10^−7^
Chinese	1,234	2,850	0.48	0.45	1.14	1.03–1.26	6.8 × 10^−3^
Meta-analysis					1.11	1.07–1.15	2.4 × 10^−8^
*Replication*							
Australian #1	145	116	0.32	0.22	1.66	1.16–2.38	5.8 × 10^−3^
Australian #2	431	567	0.27	0.24	1.22	1.00–1.48	6.2 × 10^−2^
Combined	576	683	0.29	0.23	1.32	1.11–1.58	1.7 × 10^−3^

Cont, control; OR, odds ratio. The allele frequency is for the C allele. Note that the European results show the raw allele frequencies across cohorts, with the OR calculated from logistic regression that includes covariates

### Functional relevance of GPX3 and TNIP1

Both *GPX3* and *TNIP1* are genes that could have functional relevance for ALS. The protein glutathione peroxidase 3 (GPX3), is an antioxidant molecule functionally related to superoxide dismutase 1 (SOD1)^13^; many *SOD1* single-nucleotide variants are pathogenic for ALS. In a mass spectrometric screen of sera of SOD1^H46R^ rats compared to their wild-type (WT) controls in the presymptomatic stage (12 weeks of age) of ALS, Gpx3 was detected as one of the two significant results (1.3-fold increase in expression)^14^. In the same study, Gpx3 expression was significantly lower (0.74 fold, *p* = 0.009) compared to WT controls by disease end stage, a finding which was replicated in blood sera of sporadic ALS cases (*n* = 18) and controls (*n* = 35) (GPX3 0.41-fold lower, *p* = 0.008)^14^. Both *GPX3* and *TNIP1* are functionally associated with NF-κB, the master regulator of inflammation^17, 19^, with upregulation of NF-κB associated with death of motor neurones^15^. Protein–protein interaction analysis^18^ links GPX3 to SOD1 and TNIP1 to OPTN, and *OPTN* also harbours mutations associated with familial ALS^5^. *TNIP1* is associated with a wide range of immune disorders^22, 23^, although our most associated SNP (rs10463311) is not in LD with specific SNPs associated with these disorders^24^. We investigated differential expression of *GPX3* and *TNIP1* between ALS patients and controls, but given small sample sizes, the results were not conclusive (Supplementary Note 1, Supplementary Table 3, Supplementary Fig. 6). In a pleiotropy informed analysis^25^ applied to the European GWAS summary statistics^7^, rs10463311 was identified as an ALS-associated SNP, providing additional, albeit not fully independent, support for this locus.

### Gene-based association analysis

No genes were significantly associated with ALS from gene-based association analysis implemented in fastBAT^26^ of Chinese data (based on Bonferroni correction for ~18,000 autosomal genes, significance declared at 2.8 × 10^−6^), but meta-analysed results (Supplementary Table 4) identified multiple genes (reflecting LD and overlapping gene boundaries) at the previously reported chromosome 5, 9, 14 and 17 GWAS loci. Two new loci on Chromosome 17 (17q12 and 17q21.2) were also significant (minimum genic *p* = 3.3 × 10^−7^ and 1.2 × 10^−7^, respectively). The former locus was also supported by summary statistic-based Mendelian randomization (SMR) analysis^27^ that combines the disease–SNP association with gene expression–SNP association results and has GW significance threshold of *p*_SMR_ < 8.4 × 10^−6^) (Supplementary Fig. 7; Supplementary Data 2), with most significant association for *GGNBP2* (European only *p*_SMR_ = 4.6 × 10^−6^; meta-analysis *p*_SMR_ = 9.8 × 10^−6^). The two replication samples did not provide support for the *GGNBP2* SNP implicated from the SMR analysis (Supplementary Table 5); larger sample sizes are needed to confirm the association and to provide evidence to exclude *ZNHIT3* (*p*_SMR_ = 3.1 × 10^−5^) or *MYO19* (*p*_SMR_ = 2.2 × 10^−4^) as contributing to the association in this region. Gene-set pathway analysis implemented in MAGMA and applied to the Chinese/European meta-analysis results did not find any ALS significant pathways that passed multiple testing correction (Supplementary Table 6).

## Discussion

In summary, using a cross-ethnic design we identify association of the *GPX3-TNIP1* locus with ALS. This locus was identified by combining GWAS results from our Chinese data with the largest European GWAS data^7^ and replicated in independent Australian samples. In addition, *GGNBP2* was identified using gene-based analysis and SMR analysis, although further replication is needed to confirm this result. The discovery of a novel risk locus significantly advances our understanding of ALS aetiology.

## Methods

### Chinese ALS cases and controls

The samples comprised 1,324 ALS cases and 3,115 controls. ALS cases were recruited from the Department of Neurology, the Peking University Third Hospital (Beijing, China) from 2003 to 2013. The cases were diagnosed by a neurologist specializing in ALS using the revised El Escorial criteria^28^. The controls are individuals who attended the Peking University Third Hospital, Peking University Sixth Hospital or Shanghai Changzheng Hospital (Shanghai) with no medical or family history of neurological disorders. All cases and controls are of Chinese origin from Mainland China and provided written informed consent for the study. The sample collections were approved by the ethics committees at the respective hospitals^12^. The study is compliant with the Guidance of the Ministry of Science and Technology (MOST) for the Review and Approval of Human Genetic Resources. Analyses conducted at the University of Queensland were approved by the University human research ethics committee.

### Australian replication cohort 1

ALS cases were recruited from the Royal Brisbane & Women’s Hospital (RBWH), Brisbane, Queensland and the Macquarie University Multidisciplinary Motor Neurone Disease Clinic^29^, New South Wales. The cases (*N* = 159) were diagnosed using the revised El Escorial criteria^10^. The controls are healthy individuals (*N* = 132), sourced from 4 different sites, RBWH (27 individuals), Neurology at Macquarie University, Sydney (25 individuals), the Older Australian Twin Study (OATS)^30^ comprising 90 monozygotic (MZ) twin pairs recruited in Brisbane (QIMR Berghofer Medical Research Institute (QIMR)) and Sydney (University of New South Wales (UNSW)) and Melbourne (University of Melbourne (UM)). The OATS study recruits MZ twins aged ≥65 years and were chosen for this study because the Discovery sample controls were younger than Discovery sample cases. Twin pair data helped in quality control checks but only one twin from each pair was used in analyses. The subjects provided written informed consent for the study. The study was approved by the RBWH^31^, QIMR, UNSW, UM, University of Queensland and Macquarie University Research Ethics Committees.

### Australian replication cohort 2

Patients and controls were ascertained from Macquarie University Multidisciplinary Motor Neurone Disease and Neurology Clinics, Sydney and from the Australian MND DNA bank. Patients were diagnosed with definite or probable ALS according to the revised El Escorial criteria. Patients with a family history for ALS were excluded. Control subjects were healthy individuals free of neuromuscular diseases. DNA from 471 cases and 586 controls were available for genotyping. The subjects provided written informed consent for the study. The study was approved by Macquarie University Research Ethics Committee.

### DNA extraction

In the Chinese cohort, genomic DNA was extracted from whole blood using the DNA Extraction Kit (Beijing Aide Lai Biotechnology Co. Ltd., Beijing, China). In the Australian replication cohorts, the majority of DNA was extracted from fresh whole blood using manual extraction protocols, except for 90% (118 out of 131) of UNSW/UM control samples, where DNA was extracted from frozen whole blood or lymphocytes using an automated purification system, Qiagen Autopure LS (Qiagen, Valencia, CA, USA).

### Genome-wide association study

We performed GW genotyping in the discovery cohort using the Illumina HumanOmni ZhongHua-8 v1.0 and v1.1 arrays. These arrays contain 900,015 (v1.0) and 894,517 (v1.1) variants, respectively. Before testing for the association between each variant and disease status, we carried out quality control (QC) steps to identify and exclude poor quality samples and genetic variants. We excluded individuals based on the following QC filters: (i) genotyping call rate <99% (134 individuals); (ii) sex mismatch between genotype and clinical information (6 individuals); (iii) ancestry outliers (6 SDs from HapMap-CHB means of PC1 and PC2; 30 individuals); and (iv) duplicated or related individuals (genetic relationship matrix >0.05; 195 individuals). We excluded genetic variants based on the following criteria: (i) low genotype call rate <99%; (ii) MAF <1%; (iii) deviation from Hardy–Weinberg equilibrium *p* < 10^−6^; and (iv) differential missingness in genotypes between cases and controls (*p* < 10^−6^). After these QC steps, 1,234 cases and 2,850 controls with genotypic information from 753,038 markers remained for the subsequent analyses.

We imputed unobserved genotypes into the 1000 Genomes Project Phase 1 v3 (all ethnicities) using samples and markers that passed QC. We implemented a two-step process, i.e., haplotyping using HAPI-UR^32^ and imputation using IMPUTE^33^. We imputed 38,033,906 SNPs, but after QC (i.e., excluding markers with MAF <0.01, imputation quality score <0.80 and HWE *p* < 10^−6^), 6,613,544 SNPs were available for analysis.

### Validation sample genotyping

The first validation sample was genotyped on the Illumina Human Core Exome Array. QC and imputation followed the same pipeline as for the Chinese samples. After QC, 145 cases and 116 controls were available for analysis. For the second validation sample, SNPs were genotyped via Taqman assay such that the reaction mix included 1.0 μl of genomic DNA (10 ng/μl), 0.25 μl Custom TaqMan genotyping assay 20× (Life Technologies), 2.5 μl TaqMan SNP genotyping MasterMix 2X (Life Technologies) and 6.25 μl MilliQ. The thermocycler program included 30 s at 60 °C, 10 min at 95 °C, followed by 40 cycles of 15 s at 95 °C and 1 min at 60 °C and a final step of 30 s at 60 °C. Fluorescent signals were analysed on a Viia7 Real-Time PCR System and genotypes were determined by allelic discrimination using the Viia7 Real-Time PCR System Software (Life Technologies). Genotype calling rates were 94% for rs4958872 (LD *r* ^2^ = 1 proxy for rs10463311) and 91% for rs9906189. After QC, 431 cases and 567 controls were available for analysis.

### Genetic association analysis

The association analysis between genetic variants and disease was conducted using a linear mixed model framework implemented in GCTA (mlma-loco)^34^. To compare the results, we also used a logistic regression model by fitting five principal components as covariates. Genomic inflation factor was calculated as the median of Chi-square test statistics divided by its expected value (0.455).

### Gene-based analysis

To test for the association between a set of variants within a gene (±50 kb) and ALS, we used GCTA-fastBAT^26^ with SNP association analysis *p* values as input. This test complements SNP–disease association analysis, identifying genes that may show evidence for independent associations that individually have not achieved association significance. For Chinese data analysis, we used our own GWAS data as the reference to calculate LD and ARIC samples (dbGAP accession phs000090.v1.p1) for the European sample.

### Whole-genome estimation analysis

Genomic relationship matrix (GRM) restricted maximum likelihood (GREML) analysis using GCTA^19, 35, 36^ was used to estimate the total contribution of common genetic variants on the liability of ALS or SNP-heritability. This analysis fits all SNPs simultaneously in a mixed model linear framework to estimate the proportion of variance in disease liability explained by all SNPs. To avoid bias, for example, due to common environmental factors, we excluded related individuals based on GRM values >0.05. Lifetime disease risk of 0.002 was used in the conversion of the estimate to the liability scale^37^ (compared to 0.0025 used in the European conversion, although the results are robust to these choices). LD-score regression^20^ was applied to GWAS summary statistics as an alternative method to estimate the contribution of common genetic variants to variation in the liability of ALS.

### Genetic overlap analysis

We considered estimation of the genetic correlation between ALS risk in Europeans and Chinese, using popcorn^38^ (the cross-ethnicity LDscore regression method), but calculated^39^ that the relatively small sample size for the Chinese cohort would generate an unacceptably large SE. Instead we used polygenic risk scoring (PRS) to investigate the genetic relationship between ALS in the two ethnicities. PRS were estimated for all Chinese cases and controls as the sum of risk alleles weighted by the log OR of association estimated in the European GWAS. Eight PRS were constructed for each individual using independent SNPs (based on SNPs pruned (*r* ^2^ < 0.25 in 200-kb window) that are significant at *p* value thresholds of 0.001, 0.005, 0.01, 0.05, 0.10, 0.25, 0.5 and 1. We also constructed a PRS using all SNPs without pruning for LD because of the difference in allele frequencies and LD between ethnicities. Association between the case–control status and PRS was evaluated by logistic regression. Binomial sign tests were also used to evaluate evidence of overlap in signal between Chinese and European association statistics.

### Meta-analysis

Inverse variance meta-analysis was conducted between the largest GWAS for ALS in European^7^ and our Chinese GWAS results using METAL^40^.

### In silico functional analyses

To help interpret biological function of the SNP– and gene–ALS associations, gene-set pathway analyses were performed using MAGMA^41^; this method was selected based on results of a method comparison study^42^. Gene-set pathway analyses aim to identify sets of biological pathways that are relevant to disease based on a set of disease-associated variants^42^.We also conducted SMR analysis^27^ that combines the GWAS summary statistics with gene expression association results. Here we used gene expression from blood^43^ as this is currently the largest gene expression quantitative trait loci data set. The SMR test identifies pleiotropic association of a variant that affects both the expression level of a gene and the trait. The SMR-HEIDI test attempts to determine whether the effect of the disease-associated gene on gene expression reflects a single causal variant, thus prioritizing loci for functional follow-up studies.

### Data availability

GWAS summary statistics results and gene expression data are available from http://cnsgenomics.com/data/benyamin_et_al_2017_nc/BenyaminEtAl_NatComm_Data.zip.

## Electronic supplementary material

Supplementary Information

Supplementary Data 1

Supplementary Data 2
