# Supplementary material for: Cross-ethnic meta-analysis identifies association of the GPX3-TNIP1 locus with amyotrophic lateral sclerosis
Source: Nat Commun. 2017 Sep 20;8:611. doi: 10.1038/s41467-017-00471-1 (PMC5606989; doi:10.1038/s41467-017-00471-1)
Supplement: Supplementary file 1 — Supplementary Information [file 41467_2017_471_MOESM1_ESM.pdf]

Description of Supplementary Files

File Name: Supplementary Information
Description: Supplementary Figures, Supplementary Tables, Supplementary Note and Supplementary References

File Name: Supplementary Data 1
Description: Association analysis summary statistics for those passing the genome-wide significant threshold (P_META < 5 x 10-8).

File Name: Supplementary Data 2
Description: SMR analysis results (p_SMR < 0.001).

Supplementary Table 1 . Descriptive statistics of Chinese MND GWAS data.

Parameter	Cases #1	Cases #2	Control
N Individuals	1014 cases	310 cases, 4 controls, 20 missing	3115
Genotyping Chip	OmniZhongHua -8 v1.0	OmniZhongHua-8 v1.1	OmniZhongHua -8 v1.0
N SNPs	900,015	894,517	900,015
Sex from Clinics	M:647; F:345; Unknown:22	NA	NA
Sex Imputed from Genotype	M: 638 ; F: 368; Unknown: 8	M: 200; F: 130	M: 1317; F:1788 Unknown: 10
Genotyping rate	0.988	0.996	0.993
**Quality control Stage 1**			
N Individuals removed with missingness rate > 0.01	55	0	75
N Individuals removed due to ambiguous sex	2	0	4
SNPs with missingness > 0.01	25123	37607	32181
HWE < 10-6	374	4	1221
MAF < 0.01	65279	66543	66717
N Individuals after QC	957	310	3036
N SNPs after QC	808,056	793,273	793,273
**GWAS data quality control Stage 2 after combining datasets.**			
N Individuals survived QC Stage 1	1267 cases and 3036 controls		
N SNPs survived QC Stage 1	753,042		
# Individuals removed with missingness rate >0.01	0		
GENO < 0.01	0		
HWE <10-6	2		
MAF < 0.01	0		
SNPs with differential call rate (P<10-6) in Cases and Controls after QC	2		
Genotyping rate/individual	0.999		
Individuals removed based on ethnic outliers detection	30 (15 Cases; 15 Controls)		
Individuals removed with coefficient of relatedness >0.05	193 (18 Cases; 175 Controls)		
**TOTAL Individuals survived QC**	**4,084 (1234 Cases; 2850 Controls)**		
**TOTAL SNP survived QC**	**753,038**		

HWE: P value for Hardy Weinberg Equilbrium Test. MAF: minor allele frequency

Supplementary Table 2. Test of concordance in the direction of effect between European and Chinese GWAS results given p-value thresholds.

pThreshold	pSignTest	r	pSignTest(Pruned)	r (Pruned)
0.1	1.9E-36	0.01	1.3E-01	0.011
0.05	4.4E-01	0.007	4.8E-01	-0.0028
0.001	1.0E+00	-0.074	5.0E-01	-0.052
0.0001	7.7E-01	-0.17	5.2E-03	0.017
0.00001	1.3E-01	0.064	1.2E-03	0.52
0.000001	2.2E-03	0.24	1.5E-01	0.33
0.00000005	8.5E-05	0.21	3.1E-01	-0.17

pThreshold from the European GWAS

Supplementary Table 3. Description of case and control muscle samples used in RNA-seq study.

**RNAseq samples**	**Controls***	**ALS cases**
No.	7	8
Years of age at biopsy (s.d)	54.0 ±10.2	63.6 ±10.2
Sex (F/M)	2/5	4/4
Time of biopsy since symptom onset (s.d)	NA	23 ±21 months
Onset location		
Upper limbar	NA	N=2
Lower limbar	NA	N=5
Bulbar	NA	N=1
SOD1 Positive?	NA	N=1
C9orf72 Positive?	NA	N=1

*Controls that had other forms of muscle dysfunction n=6, including focal denervation atrophy (n=2), inclusion body myositis (n=1), muscular dystrophy (n=1), selective type 2 atrophy (n=1) and active denervation with partial compensation (n=1).

Supplementary Table 4. Gene-wise significant genes ($p < 8 \times 10^{-6}$) from meta-analysis of gene-based association analysis from fastBAT analysis.

Gene	Chr	Start	End	P.EUR	P.CHN	P.META
GPX3	5	150399998	150408554	3.1E-06	0.17	2.2E-06
TNIP1	5	150409503	150467221	8.9E-07	0.36	8.5E-07
MOB3B	9	27325206	27529850	4.6E-24	0.50	8.0E-24
IFNK	9	27524311	27526496	1.6E-30	0.71	6.3E-30
C9orf72	9	27546542	27573864	6.5E-28	0.91	5.6E-27
G2E3	14	31028328	31089046	1.5E-07	0.80	2.8E-07
SCFD1	14	31091459	31205033	4.0E-09	0.17	2.7E-09
KRT18P55	17	26603011	26634408	7.0E-07	0.61	9.2E-07
TMEM97	17	26646120	26655711	6.4E-07	0.38	6.2E-07
IFT20	17	26655350	26662515	1.5E-06	0.53	1.8E-06
ZNHIT3	17	34842470	34855154	7.2E-07	0.54	8.7E-07
MYO19	17	34851598	34891305	4.7E-07	0.50	5.5E-07
PIGW	17	34891402	34895150	2.8E-07	0.51	3.3E-07
GGNBP2	17	34900736	34946276	1.1E-06	0.36	1.1E-06
LEPREL4	17	39958204	39968451	1.8E-06	0.73	2.8E-06
FKBP10	17	39968961	39979469	4.1E-07	0.58	5.3E-07
NT5C3B	17	39981333	39992523	3.6E-07	0.42	3.7E-07
KLHL10	17	39994042	40004599	2.5E-07	0.43	2.6E-07
KLHL11	17	40009798	40021629	1.3E-07	0.36	1.2E-07
ACLY	17	40023178	40075272	7.9E-07	0.56	9.8E-07

P.EUR and P.CHN are P-values for gene-based analysis in European and Chinese samples, respectively. P.META is its meta-analysis P-value.

Supplementary Table 5. The association between *GGNBP2* SNP rs9906189 chosen for replication based on SMR results.

Cohort	N Cases	N Cont	Freq Case	Freq_ Cont	OR	SE	$P_{logistic}$
**European** (Ref[2])	12,577	23,475	0.45	0.48	0.98	0.0034	2.8E-06
**Chinese**	1,234	2,850	0.38	0.39	1.00	0.010	6.7E-01
**Meta-analysis**					0.98	0.0033	4.6E-06
**Replication**							
**Australian #1**	145	116	0.53	0.46	1.31	0.17	1.2E-01
**Australian #2**	434	563	0.47	0.47	1.00	0.089	9.7E-01

Cont = Control. OR = Odds Ratio. The allele frequency is for the G allele. Note that the European results show the raw allele frequencies across cohorts, with the OR calculate from logistic regression that includes covariates.

Supplementary Table 6. Nominally significant pathways (p < 0.01) derived from GWAS meta-analysis results using MAGMA.

PATHWAY	NGENES	BETA	SE	P	P_CORRECTED
3-beta-hydroxy-delta5-steroid	4	2.05	0.53	5.0E-05	2.0E-01
cyclic-nucleotide	1	3.56	0.95	9.0E-05	3.0E-01
CDP-diacylglycerol-glycerol-3-phosphate	1	3.89	1.05	1.0E-04	3.3E-01
body	7	1.10	0.31	1.7E-04	4.5E-01
leading	2	2.30	0.70	5.0E-04	7.9E-01
heparan-alpha-glucosaminide	1	2.11	0.68	9.3E-04	9.4E-01
CP2	1	2.92	0.97	1.3E-03	9.8E-01
deoxyhypusine	2	1.28	0.43	1.5E-03	9.9E-01
Ran	16	0.55	0.19	2.3E-03	1.0E+00
melanin	8	1.05	0.38	2.8E-03	1.0E+00
natriuresis	6	0.86	0.31	3.0E-03	1.0E+00
cellular_component	472	0.11	0.04	3.3E-03	1.0E+00
amyloid	10	0.62	0.23	3.4E-03	1.0E+00
Diuresis	7	0.82	0.31	4.0E-03	1.0E+00
transketolase	2	1.79	0.70	5.5E-03	1.0E+00
Astral	1	2.65	1.05	5.7E-03	1.0E+00
BRE	2	2.23	0.88	5.9E-03	1.0E+00
L-tyrosine	1	2.57	1.02	5.9E-03	1.0E+00
enkephalin	1	2.59	1.04	6.3E-03	1.0E+00
Islet	1	2.59	1.04	6.3E-03	1.0E+00
polarisome	1	2.54	1.03	6.7E-03	1.0E+00
aminophospholipid	3	1.29	0.53	7.4E-03	1.0E+00
Spemann	1	1.90	0.79	7.8E-03	1.0E+00
oogenesis	14	0.57	0.24	9.0E-03	1.0E+00
interferon-alpha/beta	5	0.87	0.37	9.1E-03	1.0E+00
Platelet	83	0.23	0.10	9.2E-03	1.0E+00
coronary	1	1.32	0.56	9.5E-03	1.0E+00
7alpha-hydroxycholest-4-en-3-one	1	2.18	0.94	9.9E-03	1.0E+00

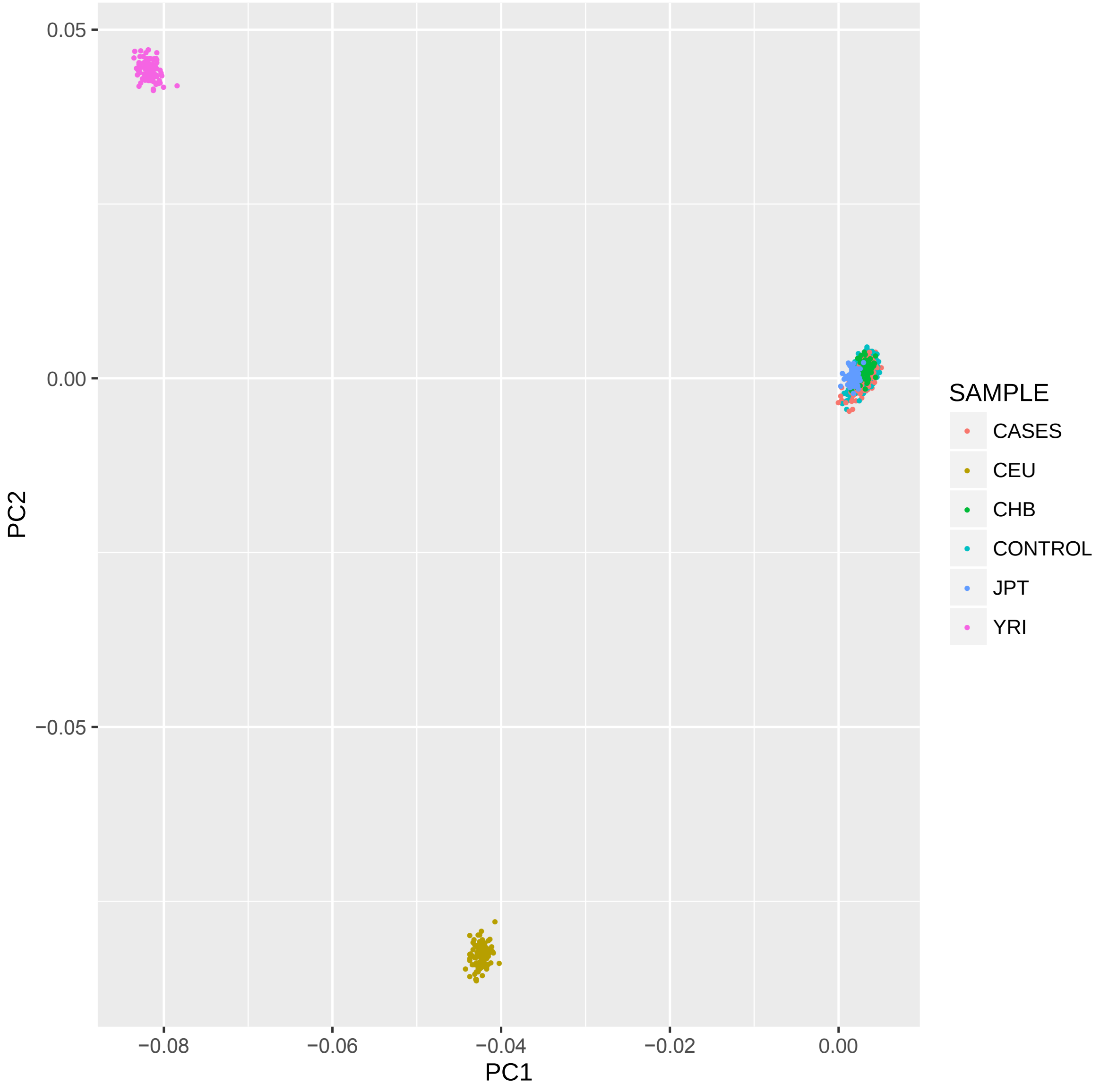

Supplementary Figure 1. Plot of principal component 1 *vs* 2 of Chinese MND samples compared to 3 major world populations (HapMap CEU, CHB/JPT and YRI). Outlying individuals (± 6SD from CHB mean) were excluded from the analyses.

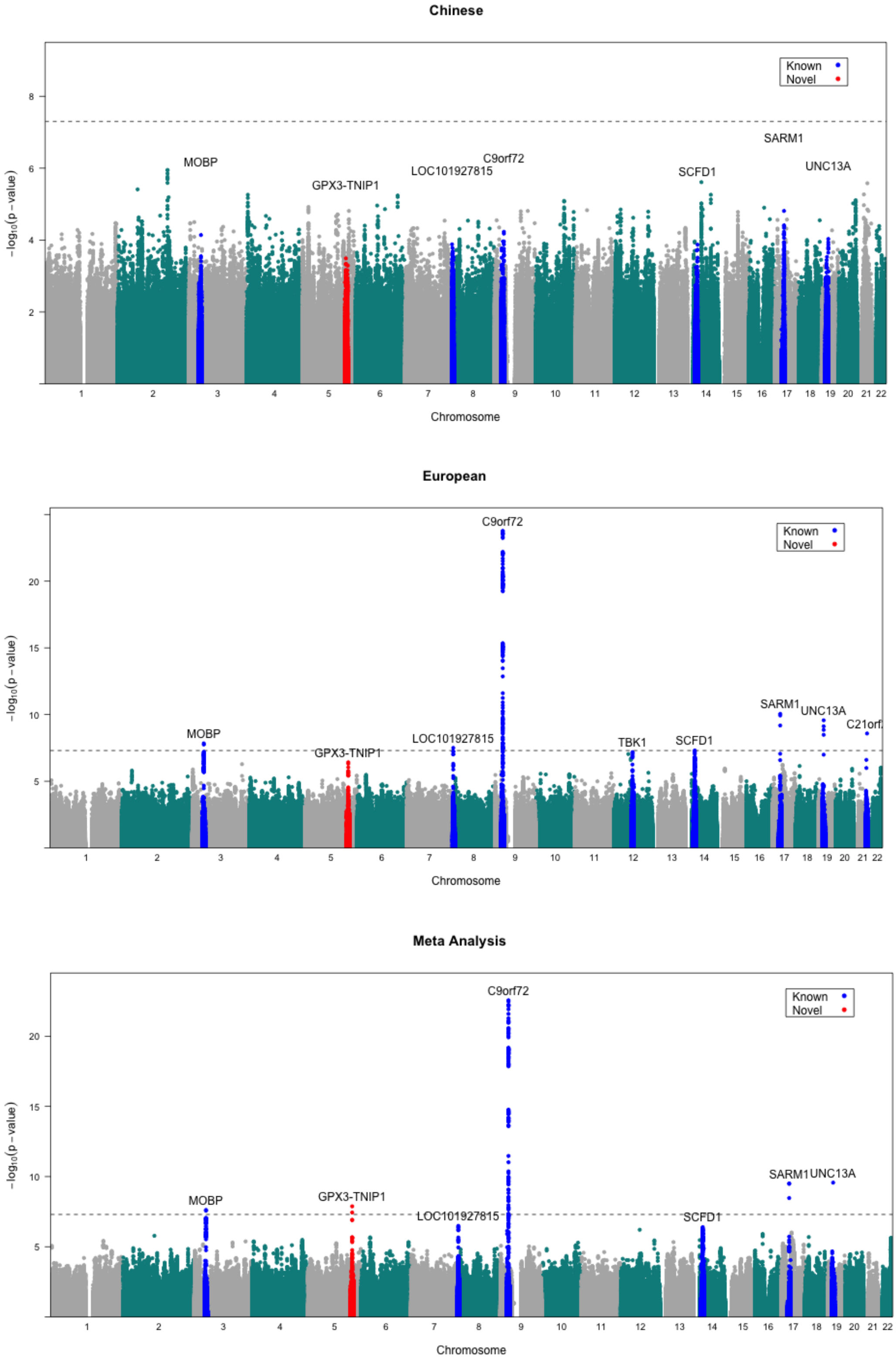

Supplementary Figure 2. Manhattan plots of a) Chinese GWAS; b) European GWAS[1] **and c) meta-analysis results between European and Chinese GWAS.**
ALS loci identified as genome-wide significant (GWS) in the meta-analysis are highlighted in blue (known) and red (novel). Two loci (*TBK1* and *C21orf2*) are not highlighted in the Chinese and meta-analysis because the top SNP and their LD friends were not available.

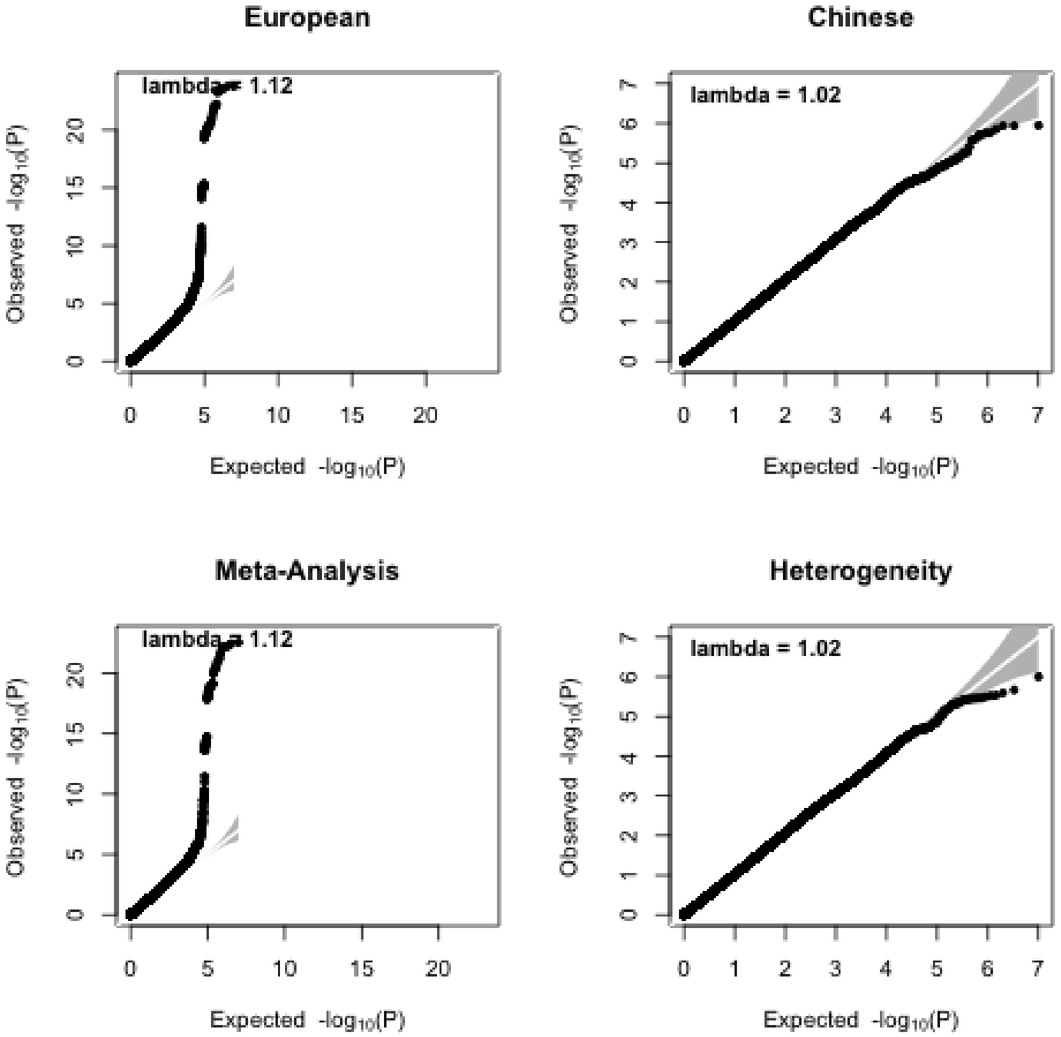

Supplementary Figure 3. QQ plots of the association –log10(P) between SNPs and ALS. The lambda$_{1000}$ for the European and Chinese GWAS are 1.007 and 1.012, respectively. The heterogeneity refers to the QQ plot of the heterogeneity –log(P) between the effect size in European compared to Chinese results.

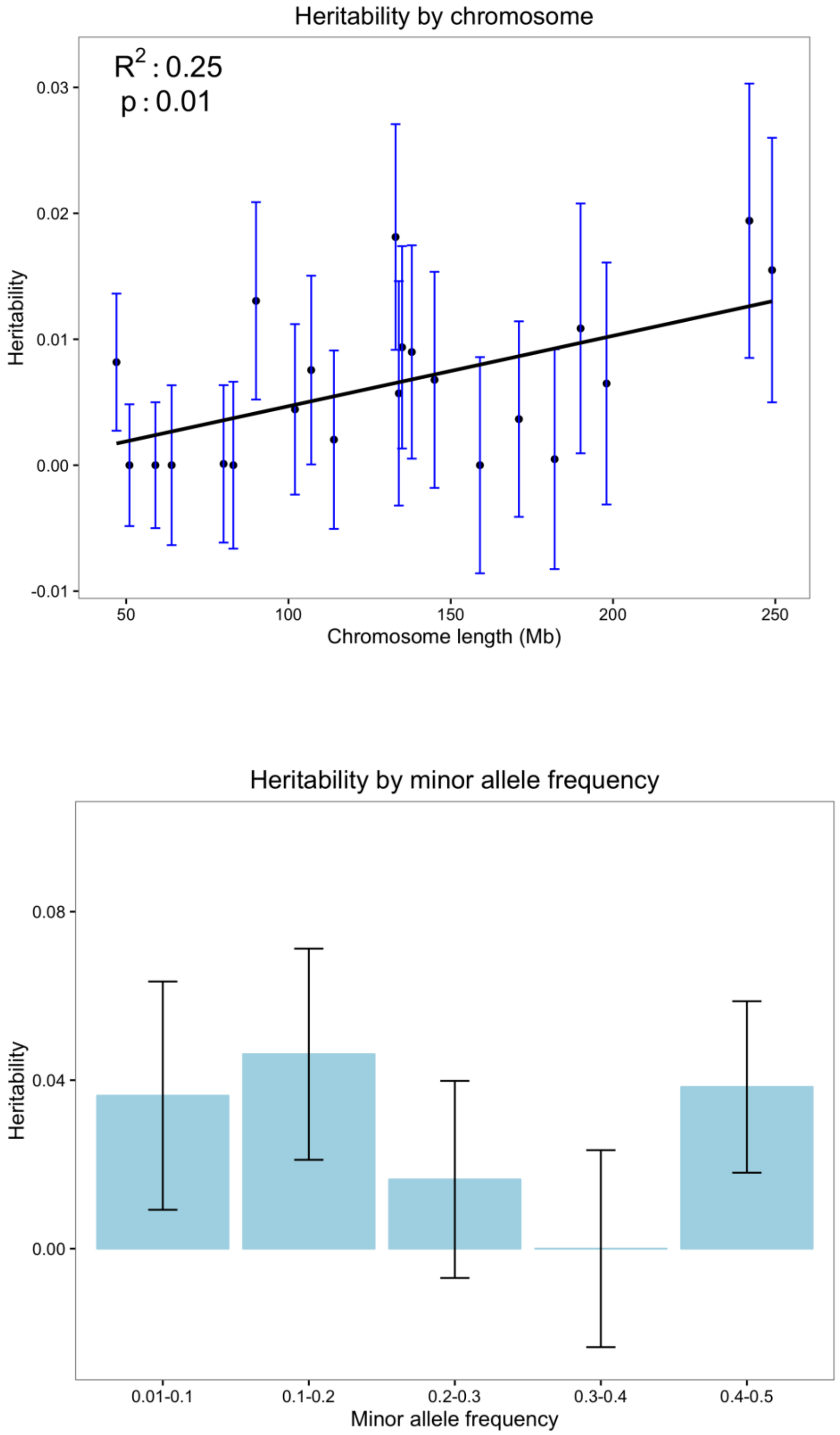

Supplementary Figure 4. The contribution of common SNPs to the proportion of variance in liability of ALS attributable to SNPs (SNP heritability) in Chinese data. The proportion of variance was partitioned into a) each chromosome and b) minor allele frequency bins. The variance was estimated using genomic restricted maximum likelihood (GREML) method implemented in GCTA software. The vertical bars are 95% confidence interval of the estimate.

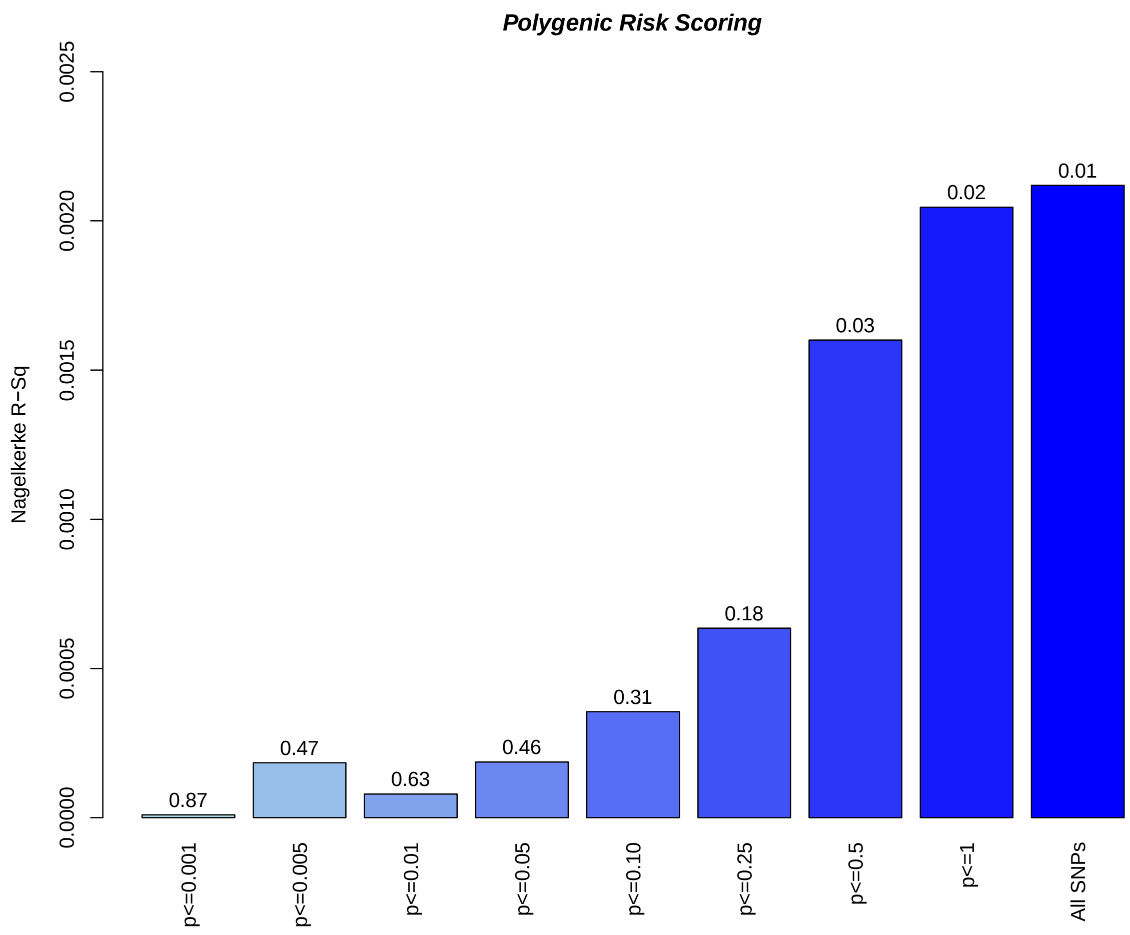

Supplementary Figure 5. Polygenic risk scoring analysis of predictors derived from European GWAS applied to Chinese data.

Polygenic risk scoring analysis showed limited evidence of predictive ability of polygenic risk score (PRS) created from European GWAS applied to Chinese data. Each bar represents the proportion of variance (Nagelkerke's R-Square) explained by polygenic score for a given subset of SNPs. The number on the top of each bar respresents the *p*-value of the general linear model association between polygenic score and disease status in Chinese samples. PRS becomes significant when we include more SNPs (ie. *p*-value threshold < 0.5 or more).

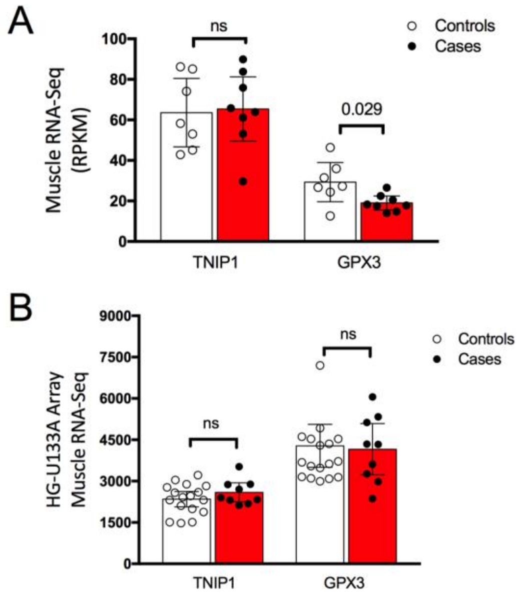

Supplementary Figure 6. RNA-seq gene expression of *GPX3* and *TNIP1* from muscle tissue (vastus lateralis).

(a) In house RNA-seq data for 8 ALS cases (red) and 7 controls (white) and (b) Publicly available Affymetrix U133A array gene expression of *GPX3* and *TNIP1* of muscle (vastus lateralis) Expression array data from 9 ALS cases (red) and 18 controls (white)[2]. To test for the difference in gene expression between cases and controls, we used a Mann Whitney U test. The vertical line in each bar represents 95% confidence interval of the mean of gene expression. The relevance of this locus to ALS was supported by these studies[3–10].

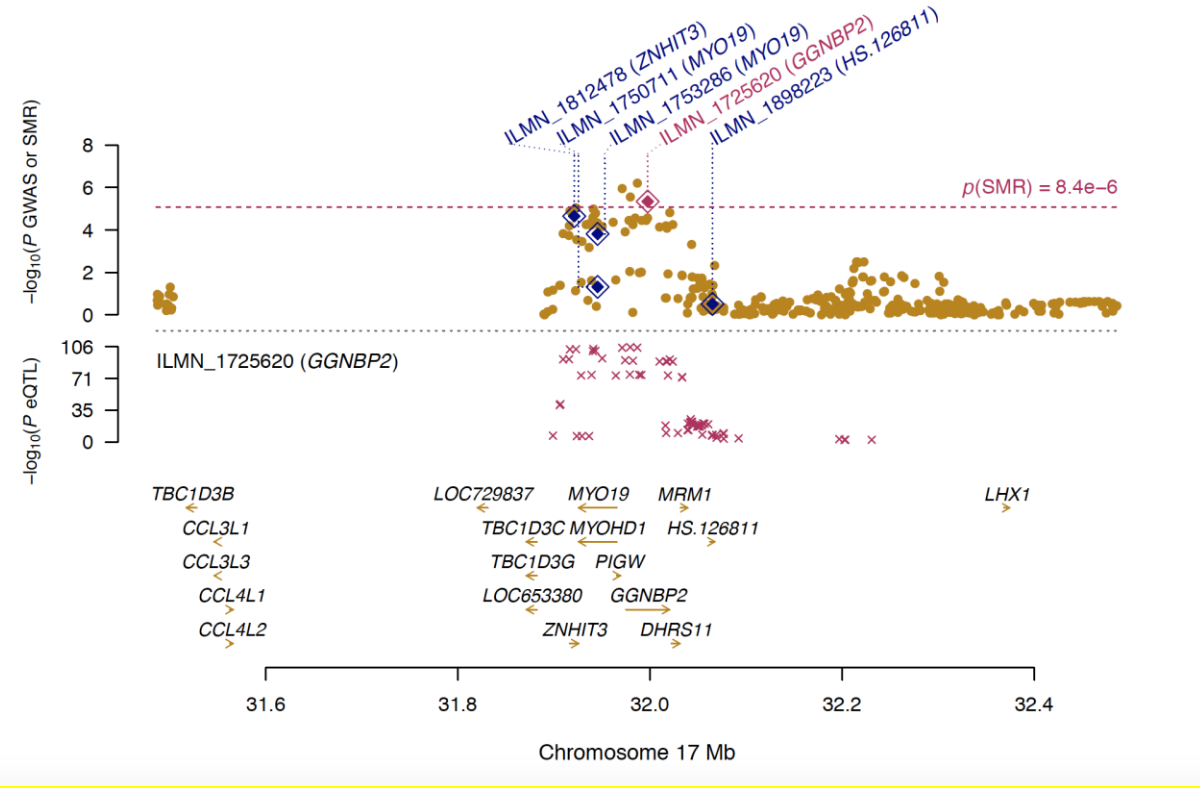

Supplementary Figure 7. Regional association plot of the most significant gene, *GGNBP2* from summary statistics-based Mendelian Randomization (SMR) analysis. Top plot, brown dots represent the European GWAS p-values, diamonds represent the p-values for probes from the SMR test. Bottom plot, the eQTL p-values of SNPs from the Westra study for the ILMN_1725620 probe tagging *GGNBP2*. The top and bottom plots include all the SNPs available in the region in the GWAS and eQTL summary data, respectively, rather than only the SNPs common to both data sets. The SNP in *GGNBP2*, rs9906189 is associated with ALS ($p=4.6 \times 10^{-6}$) and also associated with gene expression levels of a probe within *GGNBP2*, ILMN_1725620 ($p=4.07 \times 10^{-106}$).

Supplementary Note 1. Muscle gene expression

We tested for *GPX3* and *TNIP1* differential expression in muscle tissue between 8 ALS patients and 7 controls. We found that RNA expression is lower in cases compared to controls for *GPX3*, but not for *TNIP1* (**Supplementary Fig. 10a; Supplementary Table 3**). However, this finding was not supported from a publically available gene expression data set comparing 9 ALS cases with 18 controls (**Supplementary Fig 10b**). The materials and methods for muscle gene expression are described below.

Sample collection and RNA extraction. Two independent sets of muscle RNA-Seq involving ALS patients were assessed for expression changes in candidate genes identified from analyses of GWAS data, including 1) The publicly available GEO dataset (reference series: GSE3307) with biopsies typically taken from the vastus lateralis in 9 ALS cases and 18 healthy controls using the Affymetrix U133A array[2] 2) our in-house RNA-Seq data derived from patient and control muscle (vastus lateralis) biopsies collected at RBWH under approval by RBWH human research ethics committee (HREC/13/QRBW/58). Tissue was stored in 10 ml of RNALater (Life Technologies) at 4°C until received by the lab and then placed at -30°C until processed. The tissue was removed from RNALater, dabbed on tissue to remove excess RNALater and soaked in PBS for 2 minutes (this step was important – degraded RNA was obtained when this step was omitted). The tissue was weighed and muscle pieces of ~ 50 mg were added to 500 ul of Trizol reagent (Life Technologies). The muscle was homogenized using a TissueRuptor handheld rotor-stator homogenizer (Qiagen). After homogenisation an additional 500 ul of Trizol was added and the samples were mixed and incubated at room temperature for 15 minutes and centrifuged for 10 minutes at 12,000 x g at 4°C. The supernatant was transferred to a clean tube and 200 ul of chloroform added. Samples were shaken for 15 seconds before incubating at RT for 3 minutes. Samples were centrifuged for 15 minutes at 12,000 x g at 4°C. The aqueous phase was transferred to a new tube and RNA precipitated by addition of 450 ul isopropanol, 45 ul sodium acetate and 4 ul of glycogen. After incubating at room temperature for 20 minutes, samples were centrifuged for 20 minutes at 12,000 x g at 4°C. RNA pellets were washed twice in 75% EtOH followed by centrifugation for 5 minutes at 7500 x g at 4°C. The RNA was dried at room temperature and resuspended in 50 ul of RNase free water and incubated at 55°C for 15 minutes. RNA was treated with DNaseI using the TURBO DNA-free kit as per manufacturer's instructions (Ambion). Samples were quantified and quantitated on a Bioanlayser using the RNA 6000

Pico Kit (Agilent Technologies). ALS samples (n=8) all showed chronic denervation atrophy irrespective of site of onset and time since diagnosis. Available controls were age-matched (n=7) with some evidence of other muscle abnormalities (n=6) (**Supplementary Table 4**). To generate local mRNA profiles ~50 mg of the muscle biopsy was homogenised (TissueRuptor, Qiagen) and solubilized with Trizol reagent (Life Technologies, cat:15596-026) according to the manufacturer's instructions. Following TURBO™DNase treatment (Ambion, cat:AM1907) total RNA was isolated and quantitated on a Bioanlyzer using the RNA 6000 Pico Kit (Agilent Technologies). Sequencing libraries were prepared using the TruSeq Stranded Total RNA with Ribo-Zero Globin kit (Illumina) and were sequenced using an Illumina HiSeq 2000 (with V4 chemistry), with a read length of 126 bp in a paired-end sequencing mode.

RNA-Seq data analysis. CASAVA software (v1.8.2) was used to perform the image processing and demultiplex samples to generate raw sequencing reads (fastq format) for each sample. TopHat2 (v2.0.14)[11] which employs bowtie2[12] as its main aligner was used to align RNA-Seq reads to the human genome reference (hg19), with the RefSeq GTF file being provided to supply TopHat2 with a set of known gene models. HTSeq (version 0.6.1p1) was then used to quantify the gene expression level by generating a raw count table for each sample. Based on the raw count tables, edgeR (v3.8.6)[13] was used to perform the differential expression analysis between cases and controls.

Acknowledgement for ARIC study (dbGAP accession phs000090.v1.p1)

The Atherosclerosis Risk in Communities Study is carried out as a collaborative study supported by National Heart, Lung, and Blood Institute contracts (HHSN268201100005C, HHSN268201100006C, HHSN268201100007C, HHSN268201100008C, HHSN268201100009C, HHSN268201100010C, HHSN268201100011C, and HHSN268201100012C), R01HL087641,R01HL59367 and R01HL086694; National HumanGenome Research Institute contract U01HG004402; and National Institutes of Health contract HHSN268200625226C. The authors thank the staff and participants of the ARIC study for their important contributions. Infrastructure was partly supported by Grant Number UL1RR025005, a component of the National Institutes of Health and NIH Roadmap for Medical Research.

Supplementary References

1. van Rheenen, W. *et al.* Genome-wide association analyses identify new risk variants and the genetic architecture of amyotrophic lateral sclerosis. *Nat. Genet.* (2016). doi:10.1038/ng.3622
2. Bakay, M. *et al.* Nuclear envelope dystrophies show a transcriptional fingerprint suggesting disruption of Rb-MyoD pathways in muscle regeneration. *Brain* **129,** 996–1013 (2006).
3. Chi, L., Ke, Y., Luo, C., Gozal, D. & Liu, R. Depletion of reduced glutathione enhances motor neuron degeneration in vitro and in vivo. *Neuroscience* **144,** 991–1003 (2007).
4. Tanaka, H. *et al.* ITIH4 and Gpx3 are potential biomarkers for amyotrophic lateral sclerosis. *J. Neurol.* **260,** 1782–97 (2013).
5. Frakes, A. E. *et al.* Microglia induce motor neuron death via the classical NF-κB pathway in amyotrophic lateral sclerosis. *Neuron* **81,** 1009–23 (2014).
6. Oliveira-Marques, V., Marinho, H. S., Cyrne, L. & Antunes, F. Role of hydrogen peroxide in NF-kappaB activation: from inducer to modulator. *Antioxid. Redox Signal.* **11,** 2223–43 (2009).
7. Rahighi, S. *et al.* Specific recognition of linear ubiquitin chains by NEMO is important for NF-kappaB activation. *Cell* **136,** 1098–109 (2009).
8. Szklarczyk, D. *et al.* STRING v10: protein-protein interaction networks, integrated over the tree of life. *Nucleic Acids Res.* **43,** D447–D452 (2015).
9. Gateva, V. *et al.* A large-scale replication study identifies TNIP1, PRDM1, JAZF1, UHRF1BP1 and IL10 as risk loci for systemic lupus erythematosus. *Nat. Genet.* **41,** 1228–33 (2009).
10. Nair, R. P. *et al.* Genome-wide scan reveals association of psoriasis with IL-23 and NF-kappaB pathways. *Nat. Genet.* **41,** 199–204 (2009).
11. Kim, D. *et al.* TopHat2: accurate alignment of transcriptomes in the presence of insertions, deletions and gene fusions. *Genome Biol.* **14,** R36 (2013).
12. Langmead, B. & Salzberg, S. L. Fast gapped-read alignment with Bowtie 2. *Nat. Methods* **9,** 357–359 (2012).
13. Robinson, M. D., McCarthy, D. J. & Smyth, G. K. edgeR: a Bioconductor package for differential expression analysis of digital gene expression data. *Bioinformatics* **26,** 139–140 (2010).
